# Advancing skeletal health and disease research with single-cell RNA sequencing

**DOI:** 10.1186/s40779-024-00538-3

**Published:** 2024-05-30

**Authors:** Peng Lin, Yi-Bo Gan, Jian He, Si-En Lin, Jian-Kun Xu, Liang Chang, Li-Ming Zhao, Jun Zhu, Liang Zhang, Sha Huang, Ou Hu, Ying-Bo Wang, Huai-Jian Jin, Yang-Yang Li, Pu-Lin Yan, Lin Chen, Jian-Xin Jiang, Peng Liu

**Affiliations:** 1 Department of Spine Surgery, Center of Orthopedics, State Key Laboratory of Trauma and Chemical Poisoning, Daping Hospital, Army Medical University, Chongqing, 400042 China; 2 Pancreatic Injury and Repair Key Laboratory of Sichuan Province, the General Hospital of Western Theater Command, Chengdu, 610031 China; 3 Musculoskeletal Research Laboratory, Department of Orthopaedics & Traumatology, Faculty of Medicine, the Chinese University of Hong Kong, Prince of Wales Hospital, Shatin, Hong Kong SAR 999077 China; 4 Division of Plastic and Reconstructive Surgery, Department of Surgery, Stanford University School of Medicine, Sacramento, CA 94305 USA; 5 Center of Bone Metabolism and Repair, State Key Laboratory of Trauma and Chemical Poisoning, Trauma Center, Research Institute of Surgery, Laboratory for the Prevention and Rehabilitation of Military Training Related Injuries, Daping Hospital, Army Medical University, Chongqing, 400042 China; 6 Wound Trauma Medical Center, State Key Laboratory of Trauma and Chemical Poisoning, Daping Hospital, Army Medical University, Chongqing, 400042 China

**Keywords:** Skeletal disorders, Musculoskeletal system, Single-cell RNA sequencing (scRNA-seq), Cellular heterogeneity, Single cell suspension, Bioinformatic analysis

## Abstract

Orthopedic conditions have emerged as global health concerns, impacting approximately 1.7 billion individuals worldwide. However, the limited understanding of the underlying pathological processes at the cellular and molecular level has hindered the development of comprehensive treatment options for these disorders. The advent of single-cell RNA sequencing (scRNA-seq) technology has revolutionized biomedical research by enabling detailed examination of cellular and molecular diversity. Nevertheless, investigating mechanisms at the single-cell level in highly mineralized skeletal tissue poses technical challenges. In this comprehensive review, we present a streamlined approach to obtaining high-quality single cells from skeletal tissue and provide an overview of existing scRNA-seq technologies employed in skeletal studies along with practical bioinformatic analysis pipelines. By utilizing these methodologies, crucial insights into the developmental dynamics, maintenance of homeostasis, and pathological processes involved in spine, joint, bone, muscle, and tendon disorders have been uncovered. Specifically focusing on the joint diseases of degenerative disc disease, osteoarthritis, and rheumatoid arthritis using scRNA-seq has provided novel insights and a more nuanced comprehension. These findings have paved the way for discovering novel therapeutic targets that offer potential benefits to patients suffering from diverse skeletal disorders.

## Background

Skeletal disorders are a major contributor to disability-adjusted life years, affecting 1.7 billion individuals worldwide who experience degeneration, fractures, and other orthopedic conditions [1–6]. These disorders can be attributed to aging, trauma, and immune factors, impacting various skeletal components such as bones (osteoporosis, osteopenia, etc.), joints [osteoarthritis (OA), rheumatoid arthritis (RA), etc.], spines (disc degenerative disease, ankylosing spondylitis, etc.), and muscles (sarcopenia, etc.) [7]. Not only do these conditions impair the patients’ work capacity and quality of life but they also impose a significant burden on the global medical system [8, 9]. In particular, prolonged and intense training regimens can significantly contribute to the development of skeletal disorders among military personnel, potentially leading to non-combat troop reduction [10]. Therefore, there is an urgent need for a comprehensive investigation of the pathogenesis and treatment approaches for these diseases. High-resolution research strategies are required to precisely elucidate the underlying mechanisms and develop more effective therapies for skeletal diseases [11].

**Fig. 1:**
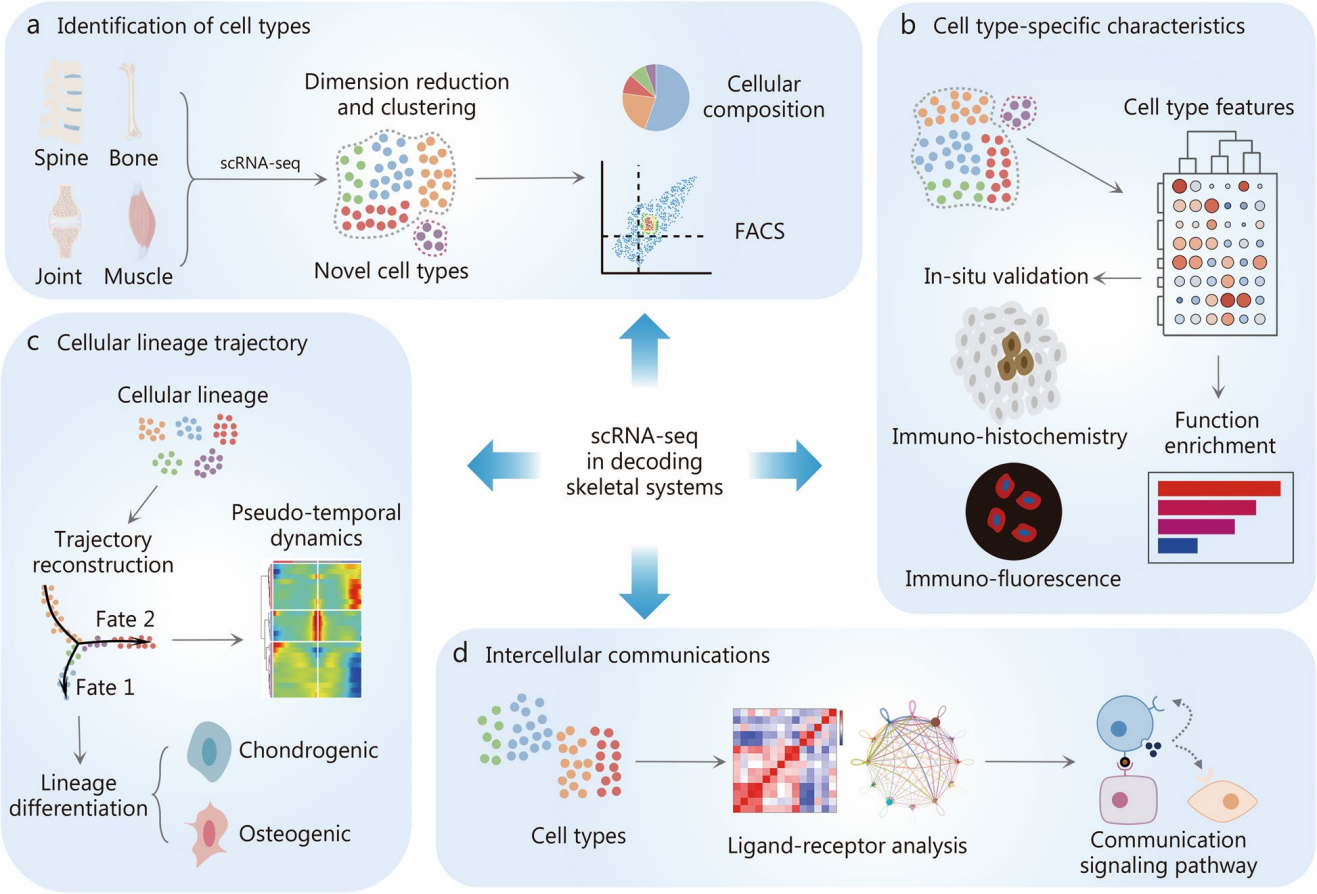
Single-cell RNA sequencing (scRNA-seq) reveals the cellular heterogeneity in unprecedented resolution in skeletal research. **a** scRNA-seq can dissect the cellular composition of specific skeletal tissues in different conditions, providing strategies of prospective isolation for target cell populations using fluorescence-activated cell sorting (FACS). **b** Differential expression analysis of scRNA-seq data helps identify both classic and novel characteristics of cell clusters. **c** The fate of cells can be predicted using single-cell trajectory inference methods, which map the developmental pathways of cells based on their gene expression profiles. **d** The relationships and intercellular communications among different cell clusters can be predicted through scRNA-seq data, which is crucial for understanding tissue function and disease progression

Over the past decade, single-cell RNA-sequencing (scRNA-seq) technology has emerged as a powerful tool for accurately examining the transcriptome at the resolution of individual cells [11–16] (Fig. 1). In contrast to bulk RNA sequencing, which provides an average measurement of gene expression across millions of cells, scRNA-seq generates sequencing libraries that map the transcriptome to individual cells, thereby clarifying the biological differences between cells [17, 18]. With its high throughput capabilities, scRNA-seq enables extensive gene profiling of more than 10^6^ single cells per run, offering the potential to identify novel cell types and characterize molecular events within cellular subpopulations [19]. In recent years, scRNA-seq has been extensively applied to study skeletal disorders, shedding light on previously unexplored aspects of the skeletal micro-world [11, 20] (Fig. 1). These approaches have allowed researchers to examine the skeletal systems at an unprecedented resolution, enhancing our understanding of cellular heterogeneity and critical cellular events that govern skeletal homeostasis and disease [21–24]. Furthermore, scRNA-seq has facilitated the elucidation of the intricate molecular network involved in intercellular crosstalk, providing crucial insights into the cellular microenvironment that often contributes to pathological processes [25–29].

Despite the numerous advantages of scRNA-seq, several challenges persist in various aspects. Firstly, it is difficult to obtain high-quality single cells from bone and cartilage tissues with dense collagen and high mineralization, often failing to meet the criteria for scRNA-seq [30]. Secondly, the diverse cell morphologies within the skeletal system necessitates careful selection of an appropriate sequencing method [30]. Lastly, inadequate bioinformatic analyses without stringent quality control measures may undermine data mining efforts. This review summarizes significant advancements in skeletal research, including sampling processing, sequencing methods, and basic bioinformatic analysis, while also presenting illustrative examples that demonstrate the full potential of scRNA-seq in skeletal studies. Additionally, the integration of scRNA-seq with multi-omic strategies may provide benefits for bridging the knowledge gaps.

## Single-cell acquisition approaches in skeletal tissues

The emergence of single-cell sequencing technology has revolutionized the field of skeletal research, providing a powerful tool to explore cellular diversity and unravel the intricate molecular mechanisms underlying complex diseases.

**Fig. 2:**
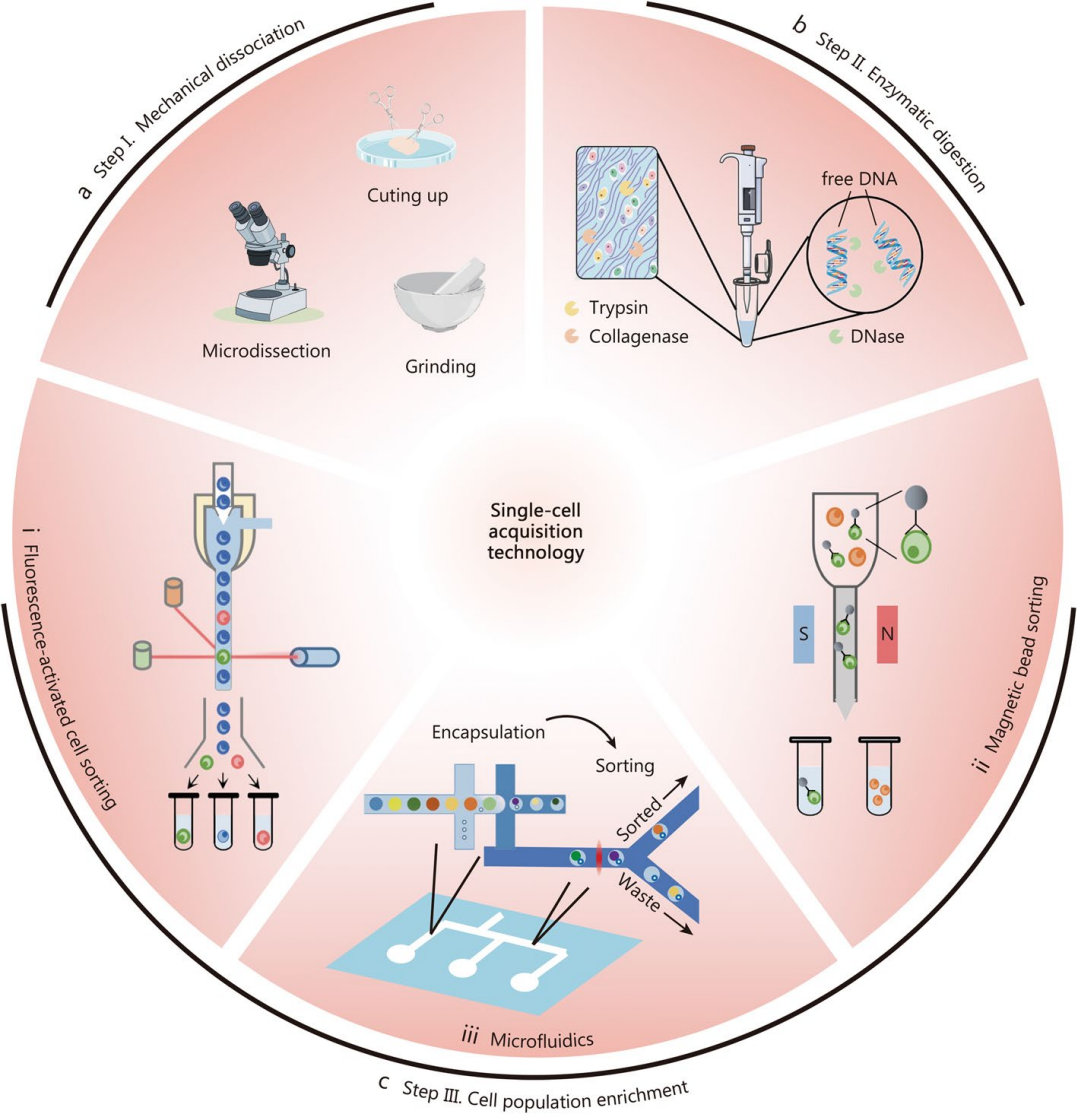
Overview of single-cell acquisition technologies used to acquire high-quality single-cell suspensions from skeletal tissues. **a** Mechanical dissociation involves techniques such as microdissection, sectioning, and grinding to physically separate cells. **b** Enzymatic digestion is an effective method that utilizes enzymes like trypsin and collagenase to break down the extracellular matrix, facilitating cell separation. DNase is also employed to extract free DNA from cell clusters. **c** (i) Fluorescence-activated cell sorting is a pivotal and precise technology for isolating single cells through fluorescence markers. (ii) Magnetic bead sorting employs magnetic beads to acquire single-cell. (iii) Microfluidic technologies represent advanced methods for single-cell acquisition. These technologies are notable for their compact design, high throughput, and enhanced sensitivity, making them indispensable in modern cellular biology

The success of single-cell sequencing technology heavily relies on high-quality techniques for capturing individual cells, which have gained significant traction in recent years [11, 31]. Numerous single-cell acquisition technologies are used to acquire high-quality single-cell suspensions from skeletal tissues, which can be divided into mechanical dissociation, enzymatic digestion, and cell population enrichment including fluorescence-activated cell sorting (FACS), magnetic bead sorting (MACS), and microfluidics [32–34] (Fig. 2). To obtain a high-quality single-cell suspension, we recommend following a streamlined approach as outlined below: (1) Mechanical dissociation. This step involves breaking down the tissue to separate the cells and enhance their accessibility for enzymatic digestion, facilitating the release of individual cells from the tissue matrix. (2) Enzymatic digestion. Following mechanical dissociation, enzymatic digestion is performed to degrade the extracellular matrix. This step is essential for separating cells from the tissue scaffold and ensuring the production of a single-cell suspension. (3) Cell population enrichment. This step employs techniques to isolate specific target cells from the heterogeneous cell suspension. Methods such as FACS or MACS can be utilized to selectively isolate desired cell populations based on molecular markers or other criteria.

### Mechanical dissociation

Mechanical dissociation is a vital technique for single-cell isolation in skeletal studies [35] (Fig. 2a). It involves microdissection to separate tissue structures, mincing using tools like razor blades and surgical scissors tailored to specific tissue types, and grinding with digestion buffer [36]. This method is crucial for preparing samples for single-cell sequencing [37], as seen in studies on bone marrow cells where it highlights a dynamic and heterogeneous molecular landscape that exhibits high responsiveness to stress [38]. Maintaining low temperatures throughout the process is essential to preserve cell integrity and protein functionality.

### Enzymatic digestion

The enzymatic digestion method uses specific enzymes to degrade the extracellular matrix of cells, effectively separating cellular components. This technique is highly efficient for single-cell separation, cost-effective, easy to operate, and minimally impairs cell integrity (Fig. 2b). For skeletal tissue with a complex extracellular matrix, the combination of collagenase and DNase I can enhance the viability of bone cell component and improve the efficiency of the separation process [36]. Collagenase digestion is the primary method applied for chondrocyte isolation by breaking down the cartilaginous extracellular matrix (ECM). Typically, three rounds of digestion lasting approximately 8 h are required to fully digest the cartilage ECM and obtain high-quality chondrocytes [39]. In the case of intervertebral discs (IVDs), a recommended combination of trypsin, protease, and collagenase is used for about 3.5 h (0.5 h for trypsin, 1 h for protease, 2 h for collagenase) to effectively break down collagen fibers and ECM to yield single cells [23]. Enzyme digestion is commonly employed as a method for isolating individual muscle or tendon cells. By incubating muscle tissue with a combination of collagenase type II, collagenase D, and dispase II for 1 h, myocytes can be effectively dissociated from other components [40].

Although the enzymatic digestion method holds promising prospects for single-cell sequencing in the skeletal system, careful consideration should be given to tissue type, enzyme combination, and enzyme concentration to determine the appropriate digestion time and ensure reproducibility and reliability. By utilizing the enzymatic digestion method, bone cell components can be obtained quickly and efficiently for subsequent analysis using scRNA-seq.

### Cell population enrichment

FACS is a critical technology for single-cell isolation, enabling precise cell sorting through fluorescence markers (Fig. 2c). It’s widely used in fields such as oncology and skeletal research, where it aids in single-cell sequencing [41–43]. For instance, Mo et al. [25] employed FACS to isolate specific skeletal cells for scRNA-seq studies on cell maintenance and lineage. However, the use of FACS may induce cellular stress that can affect sequencing accuracy, and requires specialized equipment and expertise, making it expensive and technically demanding [44]. Its resolution limitations also pose challenges in distinguishing similar cell types, with results potentially influenced by environmental and operational factors.

MACS is another widely utilized technique for single-cell acquisition, employing magnetic beads that bind to cell surface antigens or specific intracellular substances for separation in a magnetic field (Fig. 2c). This method is highly regarded for its rapidity, efficiency, and minimal cellular damage, making it the preferred choice for single-cell sequencing in skeletal research. For instance, studies have used MACS to isolate stromal cells from murine bone marrow to explore their roles in the hematopoietic stem cell niche and identify distinct subpopulations of bone marrow-derived mononuclear cells for lineage-specific investigations [45, 46]. Despite offering numerous advantages, meticulous sample preparation is necessary with MACS to prevent cellular damage and ensure precise cell sorting, and the bead binding has the potential to alter the biological properties of the cells.

Microfluidic technology is increasingly utilized in single-cell sequencing due to its compact size, high throughput, and sensitivity (Fig. 2c). It excels in the isolation, sorting, and sequencing of individual cells, facilitating efficient capture and detailed transcriptome analysis. The C1 Single-Cell Auto Prep System has been effectively employed for SMART-Seq of dendritic cells, highlighting its capability to detect transcriptomic variations and rare cellular responses [47]. Although microfluidic technology has several advantages over other single-cell acquisition methods, a critical limitation of Fluidigm microfluidic chips is their size restriction on captured cells (the largest chip is designed for cells up to 30 μm) that can undergo single-cell analysis [48].

The field of skeletal systems has been revolutionized by the advent of single-cell acquisition technology, which enables the investigation of cellular heterogeneity and intricate molecular mechanisms. Through high-quality single-cell capture technology, researchers can isolate individual cells from various sources such as bone marrow, cartilage, bones and joints. However, it is important for researchers to carefully consider both the advantages and limitations of these techniques such as reliance on cell characteristics and potential cell damage to determine the most appropriate approach for single-cell acquisition in their specific applications.

## scRNA-seq platforms applicable to the skeletal system

It is important to choose the appropriate library construction strategy for target tissues [37, 49]. Large-scale scRNA-seq methods such as the drop-seq-based 10× Genomics Chromium system, microwell-seq-based BD-Rhapsody system, and the DNBelab C4 system from MGI, can efficiently isolate numerous cells from hypercellular tissues [50–52]. For tissue with low-input cells, Smart-seq2 and CEL-seq2 are more applicable for capturing the single-cell transcriptome at a high sequencing depth using automated micropipettes or FACS [53]. In this section, we present the advantages of different strategies in terms of throughput, sequencing depth, and application scope (Table 1).

**Table 1:** Advantages and disadvantages of scRNA-seq platforms applicable to the skeletal system

Technology	Advantages	Disadvantages
10× Genomics Chromium	Large-scale; Time-effective	Probable cell loss
BD-Rhapsody	High-throughput; Tolerant for cell vitality	Limitation in cell size
Smart-seq2	High sequencing depth; The superior ability for gene detection	Low-throughput; Time and cost-consuming
CEL-seq2	High sequencing depth; Enhanced the efficiency of mRNA level measurement	Low-throughput; mRNA 3’ bias; Failure to detect non-polyadenylated transcripts

### 10× Genomics chromium system

The 10× Genomics Chromium system is one of the most widely used sequencing platforms. Specifically, cells are encapsulated in microfluidic devices and labeled using barcoding technology, leading to a significant reduction in time and cost [50, 54]. The highly parallelized nature of this system allows for the sequencing of up to 80,000 cells per sample. Moreover, its exceptional level of automation enables it to handle all aspects of the cellular suspension preparation process, single-cell capturing, library amplification, and fragment tagging within a matter of hours. Therefore, the 10× Genomics Chromium system has gained considerable popularity in skeletal research. For example, Huang et al. [55] applied this method to decode a staggering number of 102,077 cells from knee joint tissues and successfully illustrated the cellular heterogeneity associated with OA. Liu et al. [56] performed scRNA-seq on 100,987 osteosarcoma cells and revealed valuable insights into immune cell functions in recurrent and metastatic pulmonary osteosarcoma lesions. However, probabilistic capture may result in potential loss or underrepresentation of rare cell clusters, which should be taken into account.

### BD-Rhapsody system

Similar to the 10× Genomics Chromium system, the BD-Rhapsody system is also capable of efficiently handling large numbers of cells. The cell capture process in the BD-Rhapsody system is carried out using microwell precipitation, known as CytoSeq, which has over 200,000 micropores in a single plate [51]. The CytoSeq improves the probability of successful cell capture and improves the viability tolerance of cells within the BD-Rhapsody system. Moreover, the cDNAs bound to the beads can be stored at 4℃ for up to 3 months, enabling users to pool libraries from multiple samples for sequencing. For example, Tu et al. [27] applied the BD-Rhapsody system to decode human IVDs at different degenerative levels. Zhang et al. [57] utilized this technology to capture cells in bone fractures and elucidated the role of B cells in fracture repair processes. However, due to the limitation of bead binding efficiency, capturing cells with diameters greater than 20 μm may experience a significant decrease in efficiency when using the BD-Rhapsody system.

### Smart-seq2 and CEL-seq2

Smart-seq2 and CEL-seq2 are low-throughput strategies that use automated micropipettes or FACS to isolate cells into 96-well or 384-well plates [53]. These methods improve the sequencing depth of individual cells and are typically used for dissecting heterogeneity in specific cell populations [53]. Smart-seq2 captures the whole transcriptome, while CEL-seq2 only retains the sequences from the 3’ end of mRNAs, thus it is also subject to limitations due to 3’ bias [58, 59]. In addition, CEL-Seq does not detect miRNAs and other nonpolyadenylated transcripts. While this can be regarded as an advantage for enhancing mRNA level measurement efficiency, it also poses a disadvantage by failing to detect rRNA [60]. Hedlund et al. [61] employed Smart-seq2 to investigate the dynamic changes of neural stem cells after spinal cord injury based on a population of 487 GFP^+^ Nestin^+^ cells. Mizoguchi et al. [62] utilized Smart-seq2 to analyze the synovium of OA patients and identified an invasive fibroblast population located in the perivascular area of inflamed synovium. Zhang et al. [26] used CEL-seq2 to examine immune cells in the synovium of OA. In contrast to Drop-seq strategies, Smart-seq2 or CEL-seq2 can detect more genes in an individual cell. Smart-seq2, in particular, exhibits the superior ability to detect gene expression, which is particularly advantageous for cell types with low abundance transcripts such as terminally differentiated chondrocytes [63].

Given the diversity present in the skeletal tissues, it is advisable to first optimize cell isolation procedures and disentangle cellular complexity before subsequent analysis. This is especially important when conducting large-scale transcriptomic profiling and exploring rare but significant cells during bone development and related disease progression.

## Practical scRNA-seq analysis pipelines of exploring cellular heterogeneity in skeletal tissues

The raw reads obtained from Next Generation Sequencing machines undergo pre-processing, which includes data cleaning, adapter trimming, and genome mapping. These steps can be performed individually or integrated into software such as Cellranger developed by 10× Genomics. The final output is a gene matrix that is subjected to well-established analysis workflows, including Seurat (implemented in R) [64] and Scanpy (implemented in Python) [65]. While these two powerful analytic tools can manage most scRNA-seq datasets, customized analyses are required for specific projects involving various combination strategies such as differential trajectory simulation and intercellular communication networks [66, 67]. Typically, scRNA-seq enables the simultaneous revelation of transcriptomic features across all cell populations and the comprehensive prediction of their potential functions in disease progression. More importantly, targeting signature genes and enriched pathways in key cell types provides potential therapeutic targets for clinical applications. Here, we introduce the practical bioinformatic analysis steps for scRNA-seq in skeletal research.

### Quality control (QC)

QC serves as the initial step in the scRNA-seq analysis pipeline, aiming not only to eliminate low-quality data that may interfere with downstream analysis but also to exclude non-biological factors introduced by experimental conditions such as RNA degradation, elevated mitochondrial gene rates, and digestive stress. Additionally, QC can reduce noises induced by the sequencing system, such as doublets and multiplets. A rigorous QC process is crucial for filtering out low-quality cells. However, thresholds for QC should be set carefully when integrating datasets due to variations in gene expression levels among different cell types.

The basic criteria for QC involve gene numbers, count numbers, and mitochondrial gene rates. Various packages implemented in R (e.g., scuttle, DoubletFinder, DoubletDecon) and Python (e.g., scrublet, and DoubletDetection) are employed to remove potential doublets and multiplets [68–71]. After completing the QC process successfully, matrices should be normalized to correct relative gene expression abundances and facilitate cell comparison for further analysis.

### Batch effect removal

**Fig. 3:**
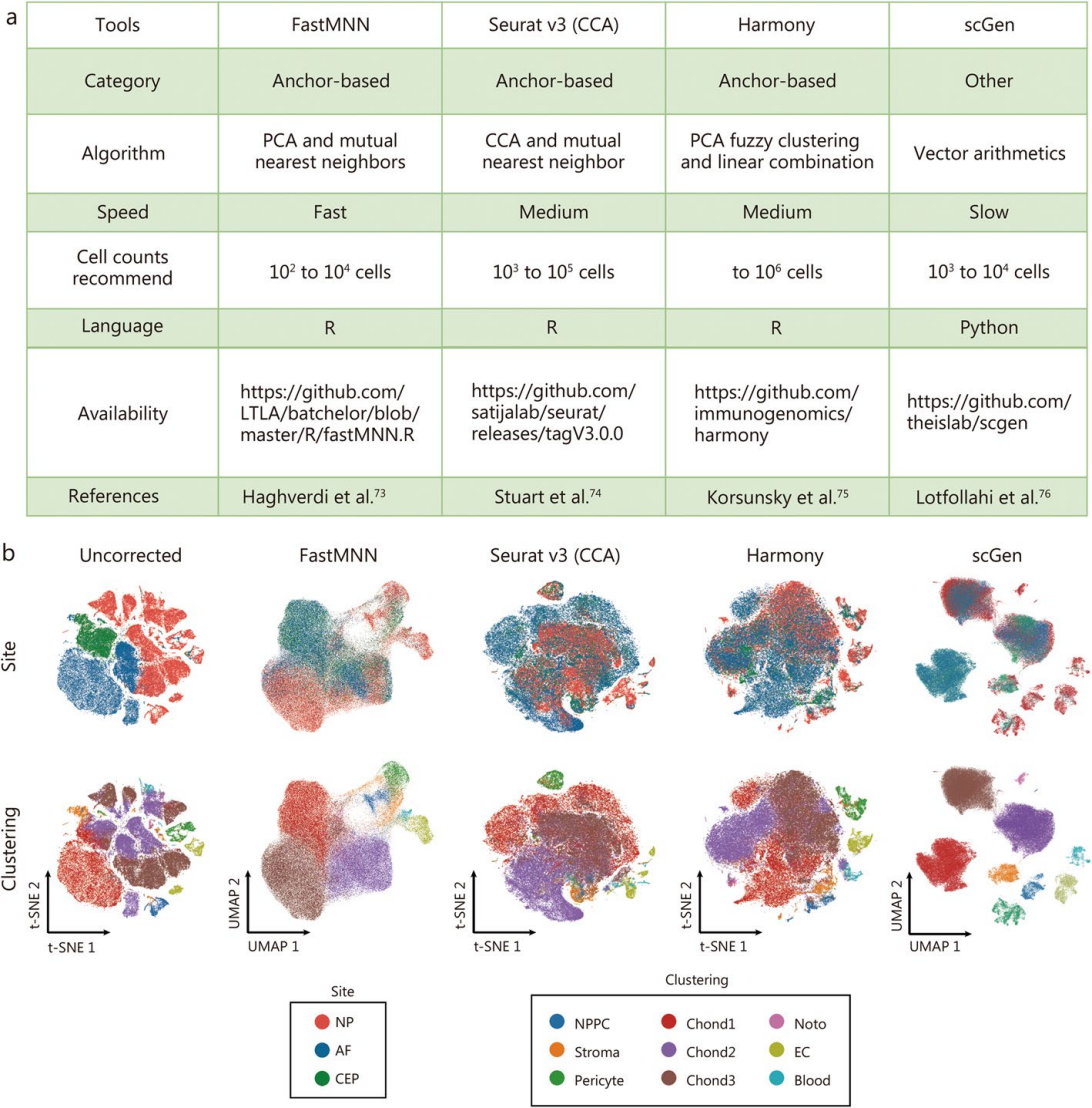
Performance of different batch effect removal strategies in the integration of scRNA-seq datasets on human IVD cells. **a** Evaluation, applicable programming language and website of FastMNN, Seurat v3 (CCA), Harmony and scGen methods. **b** The dimensionality reduction plots of raw data, FastMNN, Seurat v3 (CCA), Harmony and scGen contain two rows. In the first row, cells are colored by different sites of intervertebral disc, and in the second by cell type. Seurat v3, Harmony are embedded in t-SNE, and FastMNN, scGen are embedded in UMAP. Each method can well eliminate the batch effect while FastMNN and scGen have better performance [23]. Copyright © 2021, Published by Springer Nature. AF annulus fibrosus, Chond chondrocyte, CEP cartilaginous endplate, FastMNN fast mutual nearest neighbors, NPPC nucleus pulposus progenitor cells, t-SNE t-distributed stochastic neighbor embedding, UMAP uniform manifold approximation and projection, PCA principal component analysis, CCA canonical correlation analysis, NP nucleus pulposus, IVD intervertebral disc, Noto notochord cell, EC endothelial cell

The use of different timepoint, equipment, reagents for cell capture, and even operating personnel can inevitably lead to batch effects among datasets. More than 50 integration strategies have been reported for benchmarking scRNA-seq datasets [72, 73]. Among them, mutual nearest neighbors (MNN) or FastMNN, Seurat v3 integration, Harmony, MNN, and scGen are the most commonly used methods [74–77]. In our previous scRNA-seq datasets of human IVD cells, we compared the performances of these methods [23], and found that FastMNN and scGen showed a better balance between removing batch effect and retaining dimensional structure (Fig. 3). Huang et al. [55] applied Seurat v3 to integrate scRNA-seq datasets derived from 5 OA patients and uncovered 7 distinct populations. With the rapid increase in single-cell sequencing data on skeletal maintenance and disorders from different laboratories and platforms, effective data integration plays a crucial role in analyzing cellular heterogeneity and identifying key clusters responsible for the disease. However, it is important to avoid overcorrection during batch effect removal to preserve the biological signatures. Multiple integration methods may need to be evaluated to reveal the major features embedded in the datasets.

### Dimensionality reduction

The first step in reducing the dimensionality of high-dimensional datasets is to identify the principal components by calculating the highly variable features. To create informative and visually appealing graphs, several well-developed algorithms are used for dimensionality reduction (DR) and visualization, including principal component analysis (PCA), t-distributed stochastic neighbor embedding (t-SNE) [78], and uniform manifold approximation and projection (UMAP) [79].

PCA captures the data variance through a linear combination of gene expression levels. Its simplicity and efficiency make it usually the initial round of DR in scRNA-seq analysis, although explaining the complete polynomial relationship among features in large and complex datasets may be challenging. t-SNE and UMAP are widely used nonlinear dimensionality reduction algorithms that project the high-dimensional structural features to low-dimensional space. UMAP displays a more realistic global distribution because of its loss function and distance algorithm, whereas t-SNE retains complete local distribution information. Moreover, the UMAP algorithm has lower time consumption than t-SNE, making it faster for scaling large datasets. However, a recently developed algorithm called FFT-accelerated interpolation -based t-SNE can accelerate calculation by over 10-fold if high memory consumption on the machine is tolerable. Finally, t-SNE uses random distribution to initialize low-dimensional data, while UMAP employs the Laplace transform to assign the initial low-dimensional coordinates, resulting in higher stability of UMAP results. In orthopedic research, many studies prefer using the t-SNE reduction method to obtain a fuller composition [80–84].

### Clustering and annotation

Clustering is a crucial step in customized analysis, enabling the exploration of cellular heterogeneity and the identification of novel populations. Among various clustering methods, K-means is widely adopted due to its ability to assign each cell to the nearest center point [85]. However, being a greedy algorithm, it cannot guarantee finding the overall optimal solution, especially for clusters with rare cells [15]. In Seurat and Scanpy workflows, Louvain is a default clustering algorithm based on modularity or network graphs [86, 87]. Nonetheless, it has limitations in some situations where cluster portraits are influenced by variations in cellular distribution density within clusters [88–90]. To address these issues, Traag et al. [90] developed an optimized Leiden algorithm that offers faster running speed and ensures community connection, thereby rationalizing the process of clustering.

The strategy for defining a cluster should encompass comprehensive recognition. Typically, differentially expressed genes (DEGs) among clusters serve as signatures for each cluster. Based on this, previous studies have successfully identified various cell types in skeletal tissues [23]. Conveniently, population definition can be performed by automatic annotation packages, such as SingleR [91], Cellassign [92], Garnett [93], and scTPA [94]. It is recommended to adopt a combined strategy that considers identified marker genes and postulated cell types from annotation packages.

In many cases, the identification of a cell cluster cannot be determined by a limited number of the signature genes. Therefore, biological processes or pathways enriched by the DEGs can assist in defining cell identity. Enrichment analyses including Gene Ontology [95], Kyoto Encyclopedia of Genes and Genomes [96], and Gene Set Enrichment Analysis (GSEA) [97] are commonly employed for cluster annotation purposes. For instance, chondrocytes and fibroblasts are always associated with extracellular matrix organization, while chondrocytes exhibit closely related to the transforming growth factor-β (TGF-β) signaling pathway, and osteoblasts are linked to ossification and the secreted phosphoprotein 1 (SPP1) signaling pathway [20, 98].

Usually, known cell markers in top DEGs for each cell type are used for annotating cell clusters. Nevertheless, there may be novel genes that show significant expression levels, indicating their potential as new molecular markers for the cell cluster. These potential markers can be further validated through rigorous experiments such as in situ hybridization or immunofluorescence staining to confirm their efficacy in identifying cell types. Previous research has demonstrated that these newly identified markers can help identify key cell clusters and facilitate prospective isolation of specific cell clusters involved in skeletal development, maintenance, and disease progression [23, 99].

### Regulatory network

Gene regulatory networks (GRN) are responsible for determining and maintaining the transcriptional state of cells, making them an essential step in understanding cell states. The computational method single-cell regulatory network inference and clustering (SCENIC) is used to reconstruct GRN and evaluate their activity in each cell for identifying cell states [100]. Compared with other co-expression algorithms, SCENIC utilizes RcisTarget to identify potential direct-binding targets and exclude false positive outcomes [100]. Despite its effectiveness in analyzing specific and robust GRN underlying cell states, there are certain limitations associated with SCENIC, such as increased time-consuming when dealing with large datasets. It is advisable to infer the GRN from a subsampled dataset and include all cells during the scoring step, or opt for a more efficient algorithm such as GRNBoost [100]. Studies using SCENIC have revealed the regulators involved in enthesis progenitor cell differentiation and mineralization including SOX9 and RUNX2 [101], as well as common regulatory programs within the pathogenic subset of both articular and meniscus tissues that encompass key members of the CREB family and ZEB1 [102]. Additionally, SCENIC has been used to decipher the programs governing chondrocytes and the pericyte-like cell cluster [103]. In our previous study, SCENIC was employed to uncover the GRN that determined cell fate in the nucleus pulposus progenitor cells (NPPC) subclusters, revealing strong enrichment of SMAD3 in the PROCR^+^ NPPC-3 cluster [23]. Overall, the usage of SCENIC will provide invaluable biological insights into the mechanisms driving cellular heterogeneity.

### Differentiation trajectory inference

Pseudotime analysis is used to illustrate the lineage development or differentiation processes of specific cell types. By identifying the dynamic changes of gene expression along the trajectory, the cell state space metric can be simulated in silico. Two major tools widely used in pseudotime analysis are Monocle and RNA velocity. Monocle, proposed by Cole Trapnell et al. [104] reconstructs linear trajectories using a minimum spanning tree (MST) algorithm. RNA velocity, proposed by La Manno et al. [105], calculates the derivative of unspliced RNA to spliced RNA to obtain the kinetics of mRNA cycles and infer differentiation vectors of single cells. Commonly used RNA velocity tools include velocyto [105] and scvelo [106].

It is important to note that the MST algorithm has an advantage in trajectory construction but depends on prior knowledge when specifying the root of differentiation. In contrast, RNA velocity can predict the direction of the cell lineage based on real transcript dynamics without the knowledge of the development process. However, the result of RNA velocity largely depends on the previous dimensionality reduction diagram. He et al. [22] utilized RNA velocity to simulate chondrogenesis and osteogenesis in human embryonic skeletal development and demonstrated a subset of skeletal stem and progenitor cells that play crucial roles in the fate decision of limb bud mesenchymal differentiation. Our previous study applied Monocle3 to decipher the chondro-osteogenic potential of NPPC in human IVDs [23].

### Intercellular communications interface

The maintenance of skeletal tissues largely depends on the homeostasis of the microenvironment, which is regulated by complex intercellular communications. Therefore, analyzing these communications is critical for uncovering the molecular mechanism underlying disease. By examining the co-expression of ligand-receptor genes, it becomes possible to predict key pathways involved in regulating cell functions and identify potential targets for intervention. CellPhoneDB [107] and CellChat [108] are widely used tools in skeletal research. Wu et al. [109] applied CellPhoneDB to demonstrate that clusters of dendritic cells, T cells, and macrophages observed in RA patients display enhanced interactions mediated by molecules such as CD74, and CCL13, which may contribute to the abnormal inflammatory responses seen in RA. Ling et al. [110], using CellChat, revealed that M2 macrophages can modulate ProNPC function through macrophage migration inhibitory factor (MIF) and TGF-β signaling.

## Various applications of scRNA-seq research in skeletal health and disease

### Crucial cell clusters and molecules guiding the development and degeneration in the spine

The process of spinal development initiates with the differentiation of the sclerotome from the somite. The first pair of somites emerges around day 20 of embryonic development, followed by subsequent pairs forming at a rate of three pairs per day, which is regulated by a molecular oscillator known as the segmentation clock [111, 112]. To confirm the presence of a similar oscillator in humans, Diaz-Cuadros et al. [113] performed scRNA-seq on mouse and human pre-somatic mesoderm cells, revealing a comparable developmental trajectory and supporting the existence of a human segmentation clock. However, there is still limited understanding regarding gene expression dynamics during spinal development. Li et al. [114] conducted scRNA-seq on a pig embryo at 27-day post-fertilization, equivalent to a human embryo at approximately 9 weeks gestation. By using monocle and RNA velocity analysis methods, they constructed 2 distinct trajectories for angiogenesis and osteogenesis, while identifying 6 cell clusters. Notably, *HOXA10* expression was predominantly restricted to lumbar vertebrae clusters, suggesting its role as a determining factor in lumbar formation. Yu et al. [115] analyzed the transcriptome of human fetal spines from 8 to 17 gestational weeks at single-cell resolution and found that *HIST1H1A*^*+*^*COL2A1*^*−*^ fibroblasts may be regulated by *TUBB* along with its upstream transcription factor HOXA10. Recently, an integrated analysis combining spatiotemporal information and scRNA-seq was performed on both human and mouse spines during embryonic stages. This study also incorporated lineage tracing techniques to identify 2 types of notochord-derived nucleus pulposus (NP) cells responsible for IVD formation [116]. Collectively, the above studies provide comprehensive insights into cellular heterogeneity and regulatory processes involved in spine formation as revealed by scRNA-seq.

**Fig. 4:**
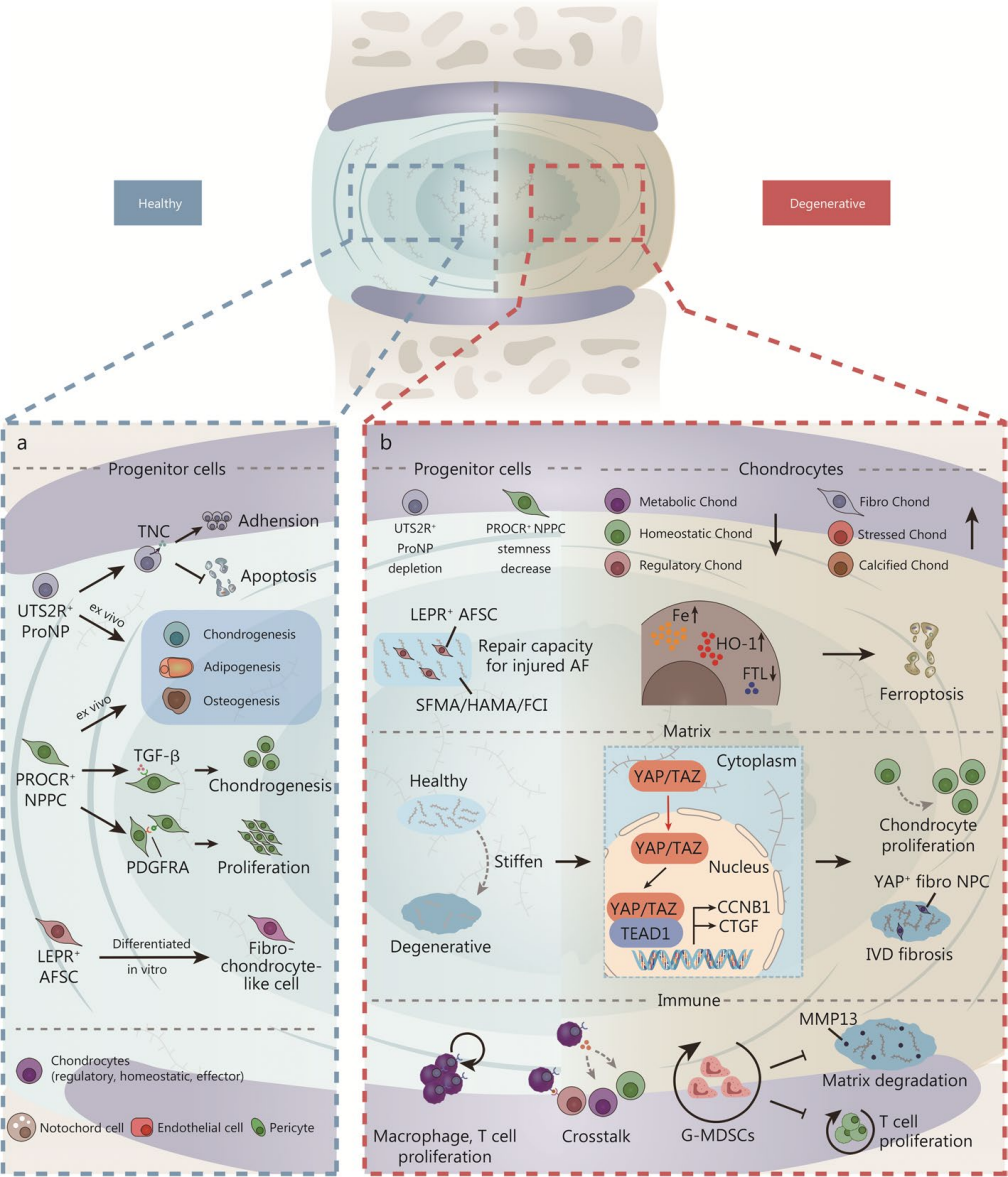
Single-cell RNA sequencing unveiled distinct cell types including progenitor cells and chondrocytes in healthy intervertebral discs (IVDs) and critical biological processes including matrix changes and immune activation during degenerative conditions. **a** In healthy IVDs, a variety of cells including progenitor cells, chondrocytes, and notochord cells play crucial roles in maintaining the IVD homeostasis. **b** In degenerated IVDs, there is a notable alteration in the phenotypes of progenitor cells and chondrocytes. The stiffening of the matrix activates YAP/TAZ signaling pathways, which in turn promotes chondrocyte proliferation and contributes to IVD fibrosis. Concurrently, macrophages and T cells not only proliferate but also engage in active crosstalk, influencing inflammatory responses. Additionally, the number of G-MDSCs increases, which plays a role in inhibiting matrix degeneration and suppressing T cell proliferation. AF annulus fibrosus, AFSC annulus fibrosus stem cell, Chond chondrocytes, NPC nucleus pulposus cell, NPPC nucleus pulposus progenitor cells, ProNP nucleus pulposus progenitors, TNC tenascin-C, TGF-β transforming growth factor-β, PDGFRA platelet-derived growth factor receptor alpha, FTL ferritin light chain, Fibro fibrogenic, MMP13 matrix metallopeptidase 13, YAP Yes-associated protein, TAZ tafazzin, TEAD1 TEA domain transcription factor 1, CCNB1 G2/mitotic-specific cyclin-B1, CTGF connective tissue growth factor, G-MDSCs granulocyte-like myeloid derived suppressor cells, UTS2R urotensin-2 receptor, PROCR protein C receptor, LEPR leptin receptor, SFMA/HAMA/FCI methacrylated SF/methacrylated HA/fibrochondrogenic inductive, HO-1 heme oxygenase-1

Following birth, the spines bear the weight of the body and the loads associated with movement. IVDs are crucial for maintaining the mechanical properties of the spine due to their elasticity and resilience. Single-cell RNA sequencing unveiled distinct cell types including progenitor cells and chondrocytes in healthy IVDs and critical biological processes including matrix changes and immune activation during degenerative conditions (Fig. 4a). Gao et al. [99] performed scRNA-seq on postnatal mouse IVDs and found *UTS2R*^+^ nucleus pulposus progenitors (ProNPs) that have trilineage differentiation potential ex vivo. They also discovered the specific expression of tenascin-C (TNC) in ProNPs and confirmed its role in promoting adhesion and inhibiting apoptosis by ex vivo studies [99]. In our previous study using scRNA-seq, we revealed various cell clusters in healthy IVDs, including chondrocytes, notochord cells, endothelial cells, and pericytes [23]. These chondrocytes were further categorized into regulatory, homeostatic, and effector subpopulations with distinct ECM properties. We also identified *PROCR*^+^*PDGFRA*^+^ NPPCs with potential stemness. Monocle3 analysis demonstrated two differentiation fates towards osteogenesis and chondrogenesis, which were subsequently confirmed ex vivo. Additionally, we used CellChat analysis to establish an intercellular communication network and identified the key regulatory molecules TGF-β and PDGFRA, which regulate NPPC chondrogenesis and proliferation. Apart from NP cells, Wang et al. [117] found *Lepr*^+^ annulus fibrosus (AF) stem cells in the intervertebral stem cell niche, expressing stemness markers like CD105. These cells differentiated into fibro-chondrocyte-like AF cells in vitro (Fig. 4a). These scRNA-seq studies have provided evidence for the presence of IVD progenitor cells along with their significant functions in maintaining homeostasis.

Spine disorders lead to severe back pain and mechanical dysfunction, significantly impairing the quality of human life. Degenerative disc disease (DDD) is a prevalent cause of low back pain [2], which is associated with microenvironmental disorders and alterations in cellular heterogeneity. Han et al. [118] conducted scRNA-seq on normal, mildly degenerative, and severely degenerative NPs, revealing an increasing inflammatory response in cartilage progenitor cells following degeneration. Similarly, Ling et al. [110] observed an inflammatory response and an increase in fibrocartilaginous NP cells, while metabolic and homeostatic NP cells decreased after degeneration in humans. Besides the enhanced inflammatory response of chondrocytes, scRNA-seq also showed an increase in *EGNL3*^*+*^ StressCs, but a decrease in *TGFBR3*^*+*^ HomCs and *GPRC5A*^*+*^ RegCs in degenerative goat IVDs, which was further validated in vitro [119]. Li et al. [120] identified inhibitory calcified chondrocytes, fibrochondrocytes, and calcified chondrocytes with high expression levels of *MGP*, *COL1A1*, and *FN1* that were more abundant within degenerative NP as well. Zhang et al. [121] found upregulated genes that related to the ferroptosis pathway in chondrocytes after degeneration. The rigid extracellular matrix represents another critical pathological feature of disc degeneration. Zhou et al. [122] revealed that matrix stiffness could activate the YAP/TEAD1-Cyclin B1 axis to promote proliferation of NP cells and IVD fibrosis, and scRNA-seq shed light on *YAP*^*+*^ Fibro NPCs as the key subcluster involved in IVD pathological fibrosis (Fig. 4b). These insights into alterations occurring in degenerative chondrocytes provided by scRNA-seq contribute to a better understanding of the mechanisms underlying function degradation seen in DDD.

As degeneration progresses, microvasculature forms when immune cells are activated and infiltrate the IVDs [123]. This phenomenon has been validated by numerous studies [27, 110, 118, 119, 124–128]. Tu et al. [27] identified granulocytic myeloid-derived suppressor cells in degenerative NP and confirmed their role in inhibiting T cells and alleviating matrix degradation in vitro. The interactions between immune cells and IVD cells at single-cell resolution have been revealed through CellPhoneDB analysis, and the successful reversal of NP ossification was achieved by inhibiting TNF-a. This was verified by Guo et al. [128] using a rat coccyx disc degeneration model. In addition, the exhaustion of stem/progenitor cells contributes to DDD. Gao et al. [99] identified *UTS2R*^+^ ProNPs that were found to be exhausted in degenerative IVDs; however, their transplantation with TNC can attenuate the progression of DDD. Wang et al. [117] established an SFMA/HAMA/FCI composite hydrogel laden with AFSC, which showed a strong repair capacity for injured AF. *PROCR*^*+*^ PC was conserved in goat IVDs and exhibited a differentiation program alteration to stemness exhaustion [119] (Fig. 4b). scRNA-seq revealed the role of immune cells in stem/progenitor cell exhaustion in DDD, providing valuable insights for potential therapeutic interventions.

Besides DDD, ankylosing spondylitis (AS) is another prevalent spine disease. Approximately 80% of patients with AS experience symptoms before the age of 30 years [129]. Although the etiology remains unknown, AS is widely considered a chronic inflammatory disease [129]. Xu et al. [130] compared the heterogeneity of peripheral blood mononuclear cells (PBMCs) from healthy individuals and AS patients, revealing an increase in naïve CD8^+^ T cells, CD8^+^ T cells, memory CD4^+^ T cells, and memory B cells in AS patients with elevated expression of genes associated with the inflammatory pathway. On the contrary, Ren et al. [131] observed a decrease in natural killer (NK) cells and a bias towards CD56^bright^ NK cells in the PBMCs from AS patients. Cribbs et al. [83] performed scRNA-seq on demethylase inhibited Th17 cells, revealing a shift from highly inflammatory cell subsets toward a resting state due to the reduced expression of key metabolic transcription factors, thereby providing a potentially effective therapeutic target for AS and other autoimmune diseases. As an autoimmune disease itself, there are strong associations between AS and Crohn’s disease [132]. Lefferts et al. [133] found that the PBMCs from patients suffering from both conditions showed a significant increase in granzyme B^+^ T cells and greater T cell maturity levels were observed. In addition, the pathological mechanism of AS involves Fibroblasts. The study by Li et al. [134] demonstrated that TNC suppressed ECM adhesion force by activating the downstream Hippo pathway signaling, which subsequently increased chondrogenic gene expression, resulting in new bone formation at entheses sites. Using scRNA-seq techniques identified a cluster of *FSP1*^+^ fibroblasts secreting TNC, thereby facilitating our understanding of AS pathogenesis [134].

Despite its advantages in spinal research, scRNA-seq has limitations including the potential loss of rare cell populations due to the low cell density in IVDs, particularly the NPs. To address these challenges, further improvements in technology and larger tissue samples for library constructions are needed.

### Cell dysfunctions contribute to the pathogenesis of OA and RA in joint

**Fig. 5:**
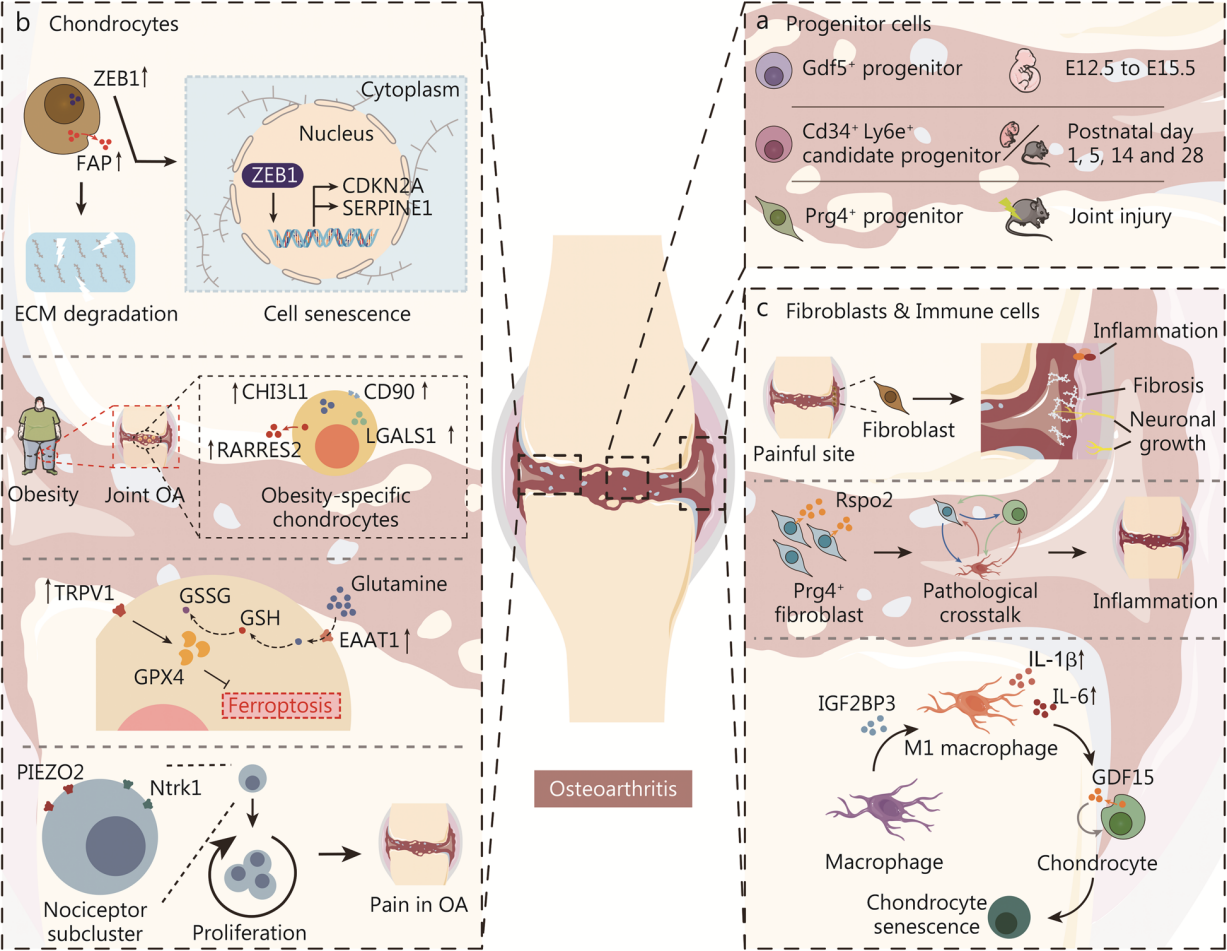
Single-cell RNA sequencing analysis elucidated critical alterations during the progression of osteoarthritis. **a** Different clusters of progenitor cells in distinguish stages of mice joints. **b** In osteoarthritis, chondrocytes undergo senescence and ferroptosis processes that contribute to cell aging and death. This cellular deterioration is associated with increased pain sensitivity in the affected joints. **c** Fibroblasts, and immune cells are triggered in osteoarthritis and improve inflammation, neuronal growth and chondrocyte senescence. OA osteoarthritis, ZEB1 Zinc finger E-box binding homeobox 1, FAP fibroblast activation protein, ECM extracellular matrix, TRPV1 transient receptor potential cation channel subfamily V member 1, GPX4 glutathione peroxidase 4, GSSG glutathione disulfide, GSH glutathione, EAAT solute carrier eamily 1 member 3, PIEZO2 piezo-type mechanosensitive ion channel component 2, Ntrk 1 high affinity nerve growth factor receptor, GDF5 growth/differentiation factor 5, CDKN2A cyclin-dependent kinase inhibitor 2 A, SERPINE1 plasminogen activator inhibitor 1, CHI3L1 chitinase-3-like protein 1, CD cluster of differentiation, RARRES2 retinoic acid receptor responder protein 2, LGALS1 galectin-1, Ly6e lymphocyte antigen 6E, Prg4 proteoglycan 4, Rspo2 r-spondin-2, IL interleukin, IGF2BP3 insulin-like growth factor 2 mRNA binding protein 3

Joints facilitate the movement and flexibility of limbs by enabling synovium development, which initiates the formation of an intermediate zone. In this zone, the Gdf5-expressing lineage actively participates in joint tissue formation and establishes a cohort of progenitor cells with co-generation capabilities [135]. To elucidate the underlying mechanism, Bian et al. [24] conducted scRNA-seq on mouse embryo knee joints and classified three clusters of Gdf5-lineage enriched cells in different developmental states through RNA velocity analysis. Pseudotime and immunofluorescence analysis further revealed the transcriptional profiles of the major developmental paths for joint progenitors [24]. Combining scRNA-seq and lineage tracking, Collins et al. [136] also found that chondrocyte injury led to lining hyperplasia, due to the proliferation and differentiation of Prg4-expressing progenitors into fibroblast-like synoviocytes. Further differentiation trajectory analysis demonstrated that Sox5 and Foxo1 were the key transcription factors of fibroblast-like synoviocytes in mice and humans [136]. Besides, Gao et al. [137] performed scRNA-seq on murine hindlimbs at postnatal day 1, 5, 14, and 28 to systematically dissect the developmental process. They identified CD34 and Ly6e-positive candidate progenitors in articular cartilage and enthesis, as well as 3 cellular developmental branches marked by *Col10a1*, *Spp1*, and *Tnni2* in the growth plate [137] (Fig. 5a). These results highlight the importance of progenitor cells in the joint formation and homeostasis.

Over time and with accumulated joint motion load, articular degenerative diseases such as OA can develop. Single-cell RNA sequencing analysis elucidated critical alterations during the progression of osteoarthritis (Fig. 5). OA is a complex condition that affects the whole joint, involving chondrocytes, fibroblasts, and immune cells in its pathogenesis [138–141]. Ji et al. [28] identified 7 distinct populations of chondrocytes in the human OA cartilage. Through GSEA, they revealed 3 novel phenotypes, effector chondrocytes (ECs), regulatory chondrocytes, and homeostatic chondrocytes, which are primarily involved in metabolism, signaling pathways, and modulating cellular homeostasis respectively. Pseudotime trajectory analysis determined potential transition among proliferative chondrocytes, prehypertrophic chondrocytes, and hypertrophic chondrocytes [28]. The identification of these cell populations greatly enhances our understanding of the pathological mechanism underlying OA. Chou et al. [142] found that intact cartilage primarily consists of homeostatic and hypertrophic chondrocyte subpopulation, while damaged cartilage is enriched with prefibrotic- and fibrotic-, regulatory-, reparative- and prehypertrophic-chondrocytes. Subsequently, Wang et al. [143] observed an expansion of CHI3L1^+^ RegCs in OA, while Li et al. [144] demonstrated the regenerative capacity of CHI3L1^+^ chondrocytes. Hu et al. [145] uncovered that fibrous cartilage degeneration is primarily induced by fibrocartilage chondrocytes, and ECs were found to predominantly exert immune function in OA. Compared to intact cartilage, the intercellular communication between different chondrocyte subclusters within damaged cartilage was enhanced thorough pleiotrophin (PTN), nicotinamide phosphoribosyltransferase (NAMPT), SPP1, TGF-β and other signaling pathways as indicated by CellChat analysis [146] (Fig. 5b). A comprehensive analysis focusing on dysfunction in these specific chondrocyte subclusters would significantly improve our understanding of the underlying cellular events contributing to OA progression.

Researchers also identified abnormal populations in OA. Swahn et al. [102] discovered a subset of genes associated with senescence that expanded in OA. Through cellular interaction analysis using CellChat, it was found to play an essential role in connecting with other cartilage clusters via ECM, TNC, and TGF-β signaling pathways [102]. The dysregulated gene *FAP* in this cluster was shown to contribute to OA pathogenesis, while upregulated ZEB1 contributed to chondrocyte senescence [102]. Wijesinghe et al. [147] identified an obesity-specific subset characterized by an inflammatory endotype. Lv et al. [148] revealed a chondrocyte cluster expressing ferroptotic hallmarks and genes preferentially. Gene set variation analysis demonstrated that TRPV1 promoted GPX4 expression to regulate chondrocyte ferroptosis, which was verified in Gpx4^+/−^ mice [148]. However, Wen et al. [149] discovered that although senescent chondrocytes hyperactivate ferroptosis, they may overexpress Solute carrier eamily 1 member 3 (SLC1A3, also known as EAAT1) to activate the glutathione system as a countermeasure against ferroptosis through metabolomics analysis. These studies suggest a close relationship between chondrocyte senescence and ferroptosis. Qu et al. [150] revealed a SPP1^+^ chondrocyte cluster exhibiting stronger angiogenic capacity and aging characteristics. Obeidat et al. [151] identified a subcluster of nociceptors co-expressing Piezo2 and Ntrk1 which is highly relevant to pain in OA (Fig. 5b). Synovitis, a common feature of OA involving active fibroblasts [139], was investigated by Nanus et al. who deciphered fibroblasts from different stages of OA at single-cell resolution [152]. Functional pathway analysis revealed that fibroblast subsets from painful sites promoted fibrosis, inflammation, and neuronal growth [152]. Knights et al. [153] found that Wnt/β-catenin signaling was overactive in post-traumatic OA (PTOA) synovium, with Rspo2 strongly induced after injury and secreted exclusively by Prg4^hi^ lining fibroblasts, further increasing pathological crosstalk and contributing to the inflammation in PTOA. Researchers are also working on elucidating the role of immune cells in the OA [110, 154–160]. Lu et al. [159] revealed IGF2BP3 as a potential macrophage mediator in silico, and verified its function of promoting macrophage M1 polarization and inflammation in vitro. Sebastian et al. [154] identified a macrophage population enriched for phagocytic genes and growth factors. Meanwhile, IL-1β could promote GDF15 expression in OA chondrocytes and induce a senescence phenotype [160] (Fig. 5c). These findings have significant implications for advancing research initiatives aimed at developing customized treatments to address specific pathological populations in OA.

**Fig. 6:**
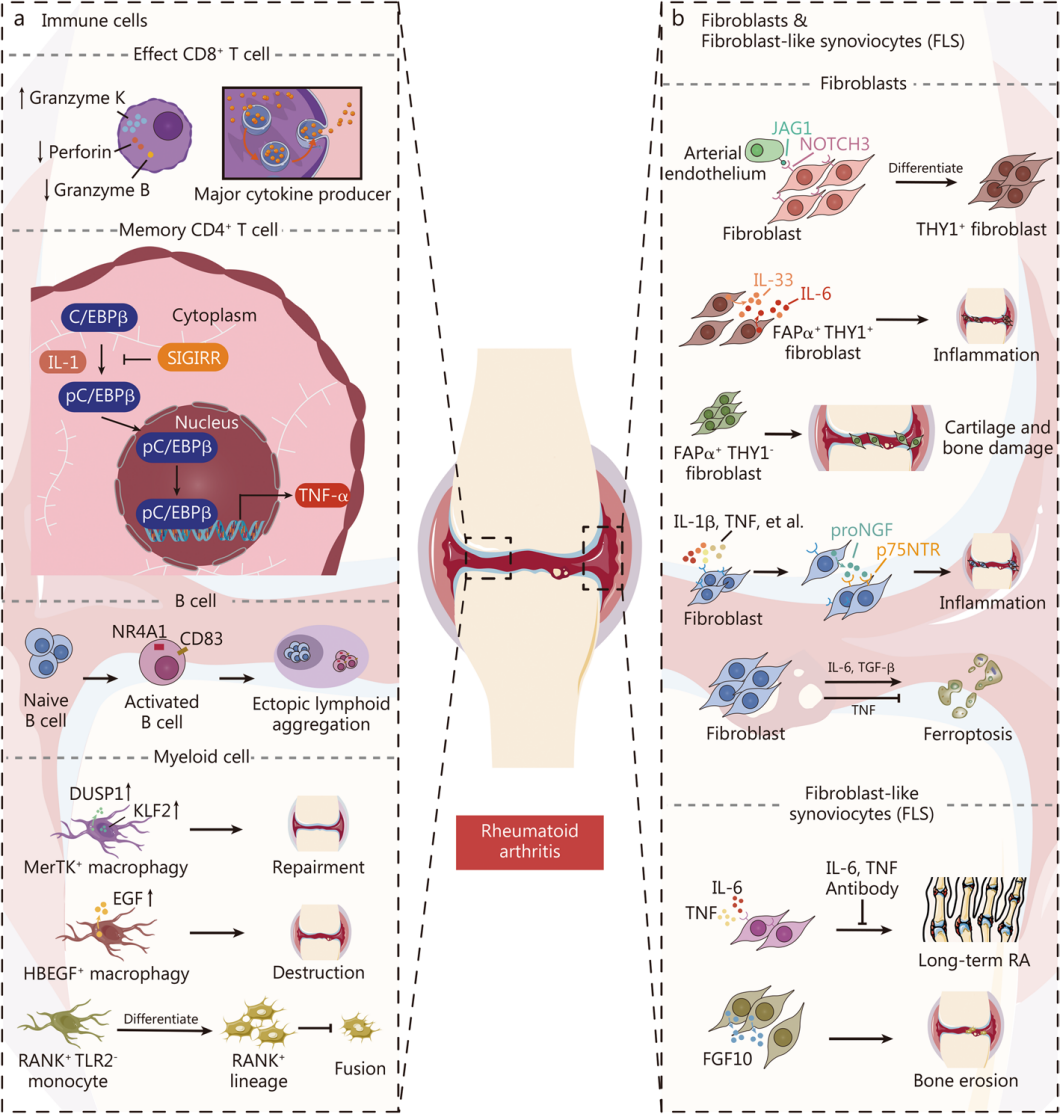
Single-cell RNA analysis unveiled the intricate roles of immune cells, fibroblasts, and fibroblast-like synoviocyte subclusters in driving inflammation, bone erosion, and other pathological processes in rheumatoid arthritis. **a** Effect CD8^+^ T cells are the major cytokine producer in RA. IL1 activates C/EBPβ phosphorylation to promote TNF production in Memory CD4^+^ T cells while SIGIRR plays an inhibitory role. Ectopic lymphoid B cells are activated in RA synovium. Myeloid cells play an important role in joint repairment and destruction. **b** Fibroblasts promote joint inflammation in RA, and FLSs drives long-term RA and contribute to bone erosion. RA rheumatoid arthritis, pC/EBPβ phosphorylated CCAAT/enhancer binding protein β, DUSP1 dual specificity protein phosphatase 1, KLF2 krueppel-like factor 2, EGF epidermal growth factor, THY1 thy-1 membrane glycoprotein, TNF tumor necrosis factor, TGF-β transforming growth factor-β, FGF10 fibroblast growth factor 10, FLS fibroblast-like synoviocyte, IL interleukin, JAG1 protein jagged-1, NOTCH3 notch homolog protein 3, CD cluster of differentiation, NR4A1 nuclear receptor subfamily 4 immunity group A member 1, RANK receptor activator for nuclear factor-κB, TLR2 Toll-like receptor 2, FAP fibroblast activation protein, proNGF nerve growth factor precursor, p75NTR p75 neurotrophin receptor, SIGIRR single immunoglobulin IL-1R-related receptor

RA is another common joint disease characterized by synovial membrane inflammation, leukocyte infiltration, and aggressive fibroblasts [4, 161, 162]. Single-cell RNA analysis unveiled the intricate roles of immune cells, fibroblasts, and fibroblast-like synoviocyte subclusters in driving inflammation, bone erosion, and other pathological processes in rheumatoid arthritis (Fig. 6). Orange et al. [163] discovered circulating CD45^−^CD31^−^PDPN^+^ preinflammatory mesenchymal cells that expand before an RA flare but decrease during exacerbation. Jonsson et al. [164] revealed that fluid CD8^+^ T cells in synovial tissue belong to an effector CD8^+^ T cell population with high expression of granzyme K and low expression of granzyme B and perforin. These cells were found to be major cytokine producers with low cytotoxic potential [164]. Argyriou et al. [165] identified 2 peripheral helper T cell states and a cytotoxic CD4^+^ T cell subset with a common differentiation pathway in the synovial fluid of RA patients at single-cell resolution. Besides, SIGIRR, preferentially expressed by memory CD4^+^ T cells, could inversely regulate RA disease activity via IL-1/C/EBPβ/TNF-α signaling axis [166]. It can be concluded that T cells play an essential role in the pathological mechanisms of RA. Moreover, ACPA^+^ and RF^+^ B cells were more abundant in the peripheral blood of RA patients and exhibited distinct transcriptional programs, implying 2 different molecular mechanisms that contribute to the increased inflammation in RA [167]. Meednu et al. [168] identified an NR4A^+^ synovial B cell population that co-expresses lymphotoxins α, β, and IL-6 and functions in ectopic lymphoid aggregation (Fig. 6a).

Myeloid cells are also tightly involved in the development of RAs [26, 169–173] (Fig. 6a). Alivernini et al. [169] identified two types of synovial tissue macrophages (MerTK^pos^TREM2^high^ and MerTK^pos^LYVE1^pos^) enriched with negative regulators of inflammation, whose potential to induce remission in RA was confirmed by their ability to elicit a reparative response in synovial fibroblasts. Conversely, HBEGF^+^ inflammatory macrophages were identified in the synovium, promoting fibroblast invasiveness through an epidermal growth factor receptor-dependent manner [170]. Zhang et al. [26] identified a cluster of proinflammatory monocytes as the major source of *IL1B* production. In addition, Zhang et al. [173] discovered a novel RANK^+^ TLR2^−^ monocyte population that negatively regulates osteoclast fusion. Although these cells can differentiate into a TRAP^+^ osteoclast lineage, they fail to undergo fusion and form osteoclasts [173] (Fig. 6a). Therefore, myeloid cells also serve as a trigger for the occurrence and development of RA.

Aggressive fibroblasts also play an important role in the progression of RA (Fig. 6b). Wei et al. [174] found that neurogenic locus notch homolog protein 3 (NOTCH3) signaling from vascular endothelial cells drives transcriptional and spatial gradients in fibroblasts. In mice, blocking NOTCH3 signaling attenuated inflammation and prevented joint damage in RA. Croft et al. [175] classified FAPα^+^THY1^+^ immune effector fibroblasts and FAPα^+^THY1^−^ bone destructive fibroblasts, while Zhang et al. [26] identified THY1(CD90)^+^HLA-DRA^high^ subliming fibroblasts as the potential major source of IL-6 in the synovium of RA patients. Chen et al. [176] identified HBEGF^+^ fibroblasts as being related to RA remission, improving our understanding of RA pathogenesis and treatment research. Farina et al. [177] discovered that the active proNGF/p75NTR axis could promote pro-inflammatory responses in synovial fibroblasts and further contribute to chronic synovial inflammation. scRNA-seq also revealed that fibroblasts are susceptible to ferroptosis, but TNF signaling promotes cystine uptake and glutathione biosynthesis to protect them from ferroptosis, as validated in vitro [178]. Thus, fibroblasts primarily contribute to RA pathology through inducing inflammation. In addition, fibroblast-like synoviocytes (FLS) are extensively involved in the mechanism underlying RA development. Smith et al. [179] found that myeloid and T cell-derived cytokines, such as TNF, IFN-γ, and IL-1β can drive 4 distinct states of FLS. Biesemann et al. [180] revealed that FLS serves as a receptor for TNF and IL-6 in OA, and the combination of TNF and IL-6 antibodies can result in sustained long-term remission in mouse models. In addition, in relapse RA patients, scRNA-seq showed that the fibroblast growth factor pathway was highly activated in lining FLS subsets and associated with bone erosions [181]. The importance of the fibroblast growth factor pathway in relapsed RA has been verified in vitro experiments and RA animal models, providing valuable insights into treatment [181] (Fig. 6b). These studies illustrated the altered function of fibroblasts, improving our understanding of the cellular basis of OA.

scRNA-seq provides a promising approach for investigating joint health and disease. However, difficulties in dissociating articular tissue such as the posterior synovium, make it challenging to identify specific pathological changes that may play critical roles in particular diseases. Therefore, it is necessary to develop more precise methods for tissue separation or utilize spatial transcriptomic analysis with single-cell accuracy.

### The roles of stem cells in bone regeneration and disease

Skeletal stem/progenitor cells are critical for maintaining the homeostatic microenvironment of bones. In 2014, Zhou et al. [182] uncovered LepR^+^ skeletal stem cells (SSCs) in the bone marrow. Moreover, Mo et al. [25] used scRNA-seq to characterize the cellular heterogeneity in LepR^+^ SSCs, discovering a quiescent Notch3^+^ subcluster associated with the vasculatures and osteo-chondrogenic differentiation via Monocle2 analysis, as well as a Sca1^+^ subcluster with high clonogenic activity. Chan et al. [183] and Worthley et al. [184] identified self-renewal and multipotent SSCs from the growth plate of newborn mice. Subsequently, SSCs were also found within the periosteum of postnatal long bones and calvaria [185]. Correspondingly, Chan et al. [186] identified human SSCs in the growth plate of 17-week-old fetal long bones. He et al. [22] further explored the emergence and features of human embryonic SSCs during early bone formation. By comparing human limb buds at 5 weeks post conception (WPC) and long bones at 8 WPC, they discovered 16 clusters, including PRRX1^+^ limb bud mesenchymal subsets and osteo-chondrogenic progenitors with differentiation potential into osteogenic and chondrogenic lineages [22]. Ambrosi et al. [187] revealed that the decline of SSCs in aged mice was connected with diminished transcriptomic diversity, which could be reversed by a combination treatment of BMP2 and the CSF1 antagonist. Yin et al. [188] revealed a Scx^+^Hoxd13^+^ musculoskeletal stem cell population through scRNA-seq analysis on E10.5, E12.5, and E15.5 murine limbs, which is indispensable for bone development. Meanwhile, Hao et al. [189] analyzed mouse hindlimb buds, postnatal long bones, and fractured long bones at single-cell resolution, and identified Cd168^+^ skeletal stem/progenitor cells (SSPCs) with highly replicating capacity and osteochondrogenic potential in embryonic and postnatal long bones. Additionally, Sivaraj et al. [80] investigated the differences between bone marrow stromal cells (BMSCs) from the metaphysis and diaphysis and demonstrated the regulatory effect of PDGFR-β signaling and the transcription factor Jun-B on BMSCs fates. The bone marrow microenvironment also plays a crucial role in regulating hematopoiesis [190]. Tikhonova et al. [38] revealed cellular heterogeneity within the bone marrow niche using scRNA-seq under stress conditions and showed an adipocytic skewing of perivascular cells. Baccin et al. [21] identified 2 Cxcl12-abundant-reticular (CAR) cell subsets (Adipo-CAR and Osteo-CAR) localized to sinusoidal and arteriolar surfaces, respectively. These subsets act as potential cytokine-secreting clusters and maintain the perivascular microenvironment.

Fractures are the most common type of bone injury, and immune cells actively participate in the process of fracture healing [191]. Zhang et al. [57] analyzed fresh and aged fracture bones of mice by scRNA-seq and identified 13 clusters. Among these clusters, B cells exhibited significant variations. Moreover, progenitor cells are essential in fracture repair. Julien et al. [192] demonstrated that skeletal muscle progenitors adopt a fibrogenic fate before engaging in chondrogenesis following a fracture. They subsequently integrated the dataset from healthy and fractured mice for 3 d post-injury, revealing a cluster of fibrochondro progenitor (FCP) primarily located at the periosteum in injured tissues [193]. Pseudotime analysis using Monocle3 uncovered FCP processing with both fibrotic and chondrogenic trajectories, highlighting the important role of FCP in fracture repair [193]. Serowoky et al. [194] found that deficient Hedgehog signaling probably leads to failed recruitment of Cxcl12-expressing SSPCs, emphasizing the importance of Shh in large-scale bone regeneration. Cxcl12^+^ BMSCs undergo identity conversion into a skeletal stem cell-like state in response to injury, which was associated with the Wnt signaling pathway [195]. On the other hand, skeletal muscle mesenchymal progenitors adopt a fibrogenic fate before engaging in chondrogenesis after fracture, which elucidates the central role played by skeletal muscle in bone regeneration [192]. Additionally, fractures can be complications arising from osteoporosis, particularly among elderly individuals [5]. Abnormal activation of osteoclasts has been closely associated with osteoporosis, and Gingival tissue-derived MSCs (GMSCs) have been found to inhibit osteoclast activity [196, 197]. Based on this finding, Wu et al. conducted scRNA-seq analysis of GMSCs and identified a CD39^+^ subcluster that specifically expressed osteogenic genes such as *BMP2* and *RUNX2*, exerting its osteogenic capacity via the Wnt/β-catenin pathway [198]. These studies provide novel insights into the mechanism of fractures and potential therapeutic targets.

Osteosarcoma (OS) is one of the most common bone malignancies worldwide, with an estimated global incidence rate of approximately 4.8/1,000,000 [199]. Guo et al. [200] found a cluster of osteosarcoma cells exhibiting highly expressing levels of collagen type VI alpha 1 chain (COL6A1), collagen type VI α 3 chain (COL6A3), and MIF, which were closely associated with lung metastasis. In recurrent OS cases, a subcluster of cancer-associated fibroblasts exhibited increased infiltration and enrichment in the epithelial-mesenchymal transition pathway [201]. Apart from OS, giant cell tumor of bone (GCTB) represents another common bone tumor that rarely leads to mortality but significantly elevates the risk of bone fractures and even disability [202]. The presence of osteoclasts and other immune cells in the tumor microenvironment plays a crucial role in these complications. Zhou et al. [203] used scRNA-seq to compare the transcriptome profiles between primary tumors, recurrent lesions, and pulmonary metastatic sites in osteosarcoma patients. They found a distinct cluster consisting of FABP4^+^ macrophages infiltrating in lung metastatic osteosarcoma lesions while observing heightened infiltration levels of osteoclast across all patients [203]. Feng et al. [204] characterized major clusters of macrophages, osteoclasts, and NK/T cells from GCTB patients and resolved the intracellular communication networks of immune cells via CellPhoneDB analysis, highlighting the role of RANK-RANKL signaling in inducing migration of osteoclasts to osteoblasts.

Despite the exceptional resolution provided by scRNA-seq, which greatly enhances our understanding of skeletal biology, certain limitations persist. Bones are primarily composed of multiple lamellar layers, resulting in a highly dense tissue structure, which may impede the effectiveness of scRNA-seq in capturing cells if the matrix is not fully disassembled. As a result, it is imperative to employ proper grinding methods and allocate sufficient time for enzymatic hydrolysis before cell separation to ensure precise and reliable outcomes.

### Regeneration of muscle and tendon in skeletal disorders

Skeletal muscle regeneration following injury relies on microsatellite cells, also known as muscle stem cells, to restore the muscular microenvironment [205]. Giordani et al. [206] depicted the cellular landscape of adult mouse hindlimb muscles and demonstrated that Scx^+^ cell clusters give rise to tenocytes, while Itga7^+^Vcam1^−^ cell clusters exhibit myogenic potential and enhance muscle stem cell (MuSC) engraftment following transplantation [206]. Andre portrayed the atlas of injured muscle and identified a novel cluster of satellite cells that might function as sensors for muscle infection or injury via the antiviral interferon pathway [207]. These atlas studies have enhanced the comprehensive understanding of the muscular ecosystem during hemostasis and repair. Penaloza et al. [208] revealed the heterogeneity of Mesp1^+^ lineage cells, which contribute to cardiac, hematopoietic, and skeletal myogenic development, and demonstrated potential differential trajectories in single-cell resolution. Moreover, Yang et al. [209] discovered a Pax3-expressing melanocyte population with robust myogenic potential, which was induced from the skin by a novel small-molecule cocktail. Although myosatellite cells are indispensable for muscle regeneration, it is also dependent on the crosstalk between MuSCs and components within their niches [210]. De Micheli et al. [211] analyzed the scRNA-seq data from hindlimb muscles of myotoxin-induced models and found that FGF2, TGF-β1, and RSPO3 regulate proliferation of myogenic stem/progenitor cell through a Syndecan-dependent mechanism. This proposed interaction network suggests a potential role for Syndecans in regulating myogenic differentiation [211]. In addition, Xi et al. [212] employed scRNA-seq to delineate the “roadmap” of human skeletal muscle and revealed the co-regulated gene networks and transcription factors that are present at distinct myogenic stages. Guo et al. [213] discovered that SRSF2 is a key regulator governing the entry of Myf5 cells into the myogenic program, ensuring their survival by preventing premature differentiation and apoptosis. Scott et al. [214] unveiled that HIC1 regulates tissue-resident mesenchymal progenitors to maintain quiescence and facilitate muscle regeneration. Epigenetically, circular RNAs also exert significant influence on muscle regeneration, as demonstrated by Yan et al. [215], who revealed that circFgfr2 regulates myogenesis and muscle regeneration through the activation of the JNK/MAPK pathway across 27 developmental stages in pig skeletal muscle.

Similar to muscle, tendons also possess the capacity to undergo self-repair. Given the importance of regulating tendon differentiation for effective self-repair, Kaji et al. [216] established directed differentiation models based on developmental cues and scRNA-seq analysis. These models successfully generated tendon and fibrocartilage cells from mouse embryonic stem cells by activating TGF-β and hedgehog pathways. They also identified retinoic acid signaling as a critical regulator of the cell fate switch between TGF-β-induced tendon and fibrocartilage lineages [216]. Moreover, tissue-resident tendon stem cells are also indispensable for tendon repair. Harvay et al. [217] revealed a cluster of *Tppp3*^+^ cells as the potential tendon stem cells, which were shown to generate new tenocytes and self-renew upon injury through lineage tracing. Fan et al. [218] found Cd9^+^Cd271^+^ tendon stem/progenitor cells characterized by nerve growth factor secretion primarily involved in the conversion from neonate to adult tendon. Fang et al. [101] demonstrated the clonogenicity and multipotency of Gli1-expressing progenitors, which function as stem cells during tendon regeneration, while Harvey et al. [217] found that PDGFRA-expressing *Tppp3*^+^ tendon stem cells were regulated by PDGFR-AA to produce new tenocytes. By combining single-cell gene regulatory network analysis, in vitro inhibitor identification, and in vivo deletion of specific genes related to tendons, Fan et al. [218] verified that the SHP2 signaling pathway is a crucial regulator for tendon maturation. Furthermore, the tendon microenvironment plays a crucial role in influencing tendon repair. Muscat et al. [219] identified the presence of macrophages and T cells in adult tendons using scRNA-seq, which contributes to the homeostasis in tendons. Following the knockout of chemokine C-C-motif receptor 2 (CCR2), a key molecule for macrophage recruitment, there was an observed decrease in myofibroblast and impaired functional recovery during the later stages of healing [219].

## Challenges and prospects

In the developing field of musculoskeletal research, the advent of scRNA-seq has ushered in a new era, enabling researchers to delve into the intricacies of the transcriptome with unprecedented precision at the single-cell resolution. This powerful tool has made significant contribution to elucidating the underlying mechanism of various diseases. Nevertheless, scRNA-seq faces several challenges that must be addressed when applied in skeletal system research.

Firstly, the sheer volume of data generated by scRNA-seq poses a formidable challenge, as it produces vast amounts of high-dimensional data that complicates the extraction of information with biological importance. Advanced bioinformatic analysis can help pinpoint key factors amidst the data explosion. For example, Cell BLAST, developed by Cao et al. [220], empowers the precise and swift retrieval and annotation of newly generated single-cell data within existing databases, thereby enhancing the overall accuracy and efficiency of the annotation process. To tackle the challenge posed by high-dimensional data processing, it becomes imperative to delve deeper into the intricacies of this issue. The future may witness the development of more sophisticated machine learning algorithms for constructing more accurate prediction models and innovative dimensionality reduction techniques to select more vital factors for analysis. These developments will empower researchers to extract biologically significant information from complex datasets more efficiently. It is essential to recognize that these findings must be verified by rigorous biological experiments to clarify their biological validity and clinical relevance.

Secondly, the lack of comparability among study outcomes in skeletal research arises from the heterogeneity in sampling standards, sequencing methods, and analysis approaches that have been widely employed in scRNA-seq. Moreover, difficulties in data integration due to negative data-sharing behaviors greatly diminish the value of resources in studies. This challenge can be attributed to the absence of standardized practices across laboratories. To overcome this obstacle, future endeavors should focus on establishing clear standards and specifications that ensure data consistency across different research settings. Initiatives aimed at creating universally accepted standardization processes and fostering shared data principles are essential milestones for advancing the field. Encouraging multi-center collaborations emerges as another paramount strategy to enhance the reliability of scRNA-seq applications in skeletal research. By promoting a collective commitment to standardized practices, researchers can not only ensure the reproducibility of their findings but also facilitate robust data sharing. Such collaborative efforts are pivotal for realizing the full potential of scRNA-seq by enabling more reliable, comparable, and impactful results that transcend individual research boundaries. Ultimately, the establishment of a shared framework will amplify the collective impact of research endeavors and accelerate progress in musculoskeletal studies.

In addition to scRNA-seq, a plethora of emerging single-cell sequencing technologies are poised to revolutionize our understanding of skeletal research, offering more nuanced insights into cellular dynamics and molecular mechanisms. Single-cell ATAC-seq, for instance, presents a groundbreaking approach by unraveling chromatin accessibility profiles at the single-cell level [221]. The approach that uses transposase to capture chromatin openness provides researchers with the opportunity to employ high-throughput sequencing for an in-depth examination of chromatin accessibility and epigenetic properties [221]. Not only does this technique provide intricate details about the accessibility of genomic regions but also sheds light on the complex landscape of transcriptional regulation within individual cells. The ability to discern chromatin accessibility offers a valuable complement to scRNA-seq, enabling researchers to delve deeper into the epigenetic underpinnings of skeletal processes. Spatial transcriptome, another emerging frontier, addresses a critical limitation of scRNA-seq by restoring spatial context to gene expression patterns [222]. It employs either gene chips or image-based technologies to transform the gene expression information from sampled sites into digital signals [222]. By visualizing gene expression and distribution within tissue sections, spatial transcriptomics enables researchers to discern how cells interact in their native microenvironment. This capability provides a more holistic understanding of the spatial organization of cell populations within skeletal tissues. Multi-omics approaches that integrate genomics, metagenomics, transcriptomics, proteomics, and metabolomics offer a more comprehensive and insightful perspective on the pathological mechanism in orthopedic diseases [223]. Furthermore, scRNA-seq brings plenty of clues for the theoretical basis of utilizing genetic models and allows for the integration of clonal relationships into these molecular landscapes [224]. Sarah Bowling et al. [225] introduced the CRISPR array repair lineage tracing mouse line and accompanying analysis tools, which enable the simultaneous investigation of lineage and transcriptomic information in individual cells in vivo. A novel sequencing approach called Camellia-seq has recently emerged, allowing for the concurrent measurement of chromatin accessibility, DNA methylation, gene expression, and lineage information within individual cells [226]. Coupled with Cas9-TdT CRISPR array repair lineage tracing (DARLIN), Li et al. [226] designed a stable inducible lineage-labeling genetic mouse model capable of labeling approximately 10^18^ gene sites, and used Camellia-seq to systematically unveil unprecedented insights into the cellular fate decision process at the single-cell level. As we embrace these advancements, the combined application of scRNA-seq with these emerging technologies promises to unveil a more detailed and interconnected landscape of skeletal biology. These innovative tools not only complement the limitations of scRNA-seq but also pave the way for a more holistic and integrative exploration of the complexities inherent in musculoskeletal research.

## Conclusions

In this review, we outlined the essential steps for acquiring high-quality single-cell suspensions from skeletal tissues, discussed the commonly employed scRNA-seq platforms in skeletal system research, and elucidated the indispensable bioinformatic analysis pipelines crucial for deciphering cellular heterogeneity and responses in skeletal homeostasis and diseases. These cutting-edge technologies hold promise in our pursuit of a comprehensive understanding and effective management of skeletal disorders. By harnessing the power of scRNA-seq technology, we can effectively address current challenges in skeletal research, enhance our understanding of the underlying mechanisms driving relevant diseases, propel precision medicine advancements in this field, and ultimately contribute to the prevention and treatment of skeletal disorders in military medicine.

## Data Availability

The datasets analyzed during the current study are available in the GEO repository (GSE160756).

## Abbreviations

AF: Annulus fibrosus
AS: Ankylosing spondylitis
BMSCs: Bone marrow stromal cells
CAR: Cxcl12-abundant-reticular
COL6A1: Collagen type VI alpha 1 chain
COL6A3: Collagen type VI alpha 3 chain
DARLIN: Cas9-TdT CRISPR array repair lineage tracing
DDD: Degenerative disc disease
DEGs: Differentially expressed genes
DR: Dimensionality reduction
ECM: Extracellular matrix
ECs: Effector chondrocytes
FACS: Flow cytometry cell sorting
FCP: Fibrochondro progenitor
FLS: Fibroblast-like synoviocyte
GCTB: Giant cell tumor of bone
GMSCs: Gingival tissue-derived MSCs
GRN: Gene regulatory network
GSEA: Gene set enrichment analysis
IVD: Intervertebral disc
MACS: Magnetic bead sorting
MIF: Migration inhibitory factor
MNN: Mutual nearest neighbors
MST: Minimum spanning tree
MuSC: Muscle stem cell
NAMPT: Nicotinamide phosphoribosyltransferase
NK: Natural killer
NOTCH3: Neurogenic locus notch homolog protein 3
NP: Nucleus pulposus
NPPC: Nucleus pulposus progenitor cells
OA: Osteoarthritis
PBMCs: Peripheral blood mononuclear cells
ProNPs: NP progenitors
PTN: Pleiotrophin
QC: Quality control
RA: Rheumatoid arthritis
SCENIC: Single-cell regulatory network inference and clustering
scRNA-seq: Single-cell RNA sequencing
SPP1: Secreted phosphoprotein 1
SSCs: Skeletal stem cells
SSPCs: Skeletal stem/progenitor cells
TGF-β: Transforming growth factor-β
TNC: Tenascin-C
t-SNE: t-distributed stochastic neighbor embedding
UMAP: Uniform manifold approximation and projection
WPC: Weeks post conception
